# Senescent Alveolospheres: A Preliminary 3D Model for Exploring Epithelial Senescence and Pro-Fibrotic Signaling

**DOI:** 10.3390/ijms27146171

**Published:** 2026-07-10

**Authors:** Aurora Longhin, Valentina Gatta, Gabriella Teti, Mirella Falconi

**Affiliations:** 1 Department of Biomedical and Neuromotor Sciences (DIBINEM), University of Bologna, 40126 Bologna, Italy; aurora.longhin2@unibo.it (A.L.); valentina.gatta6@unibo.it (V.G.); 2 Department of Medical and Surgical Sciences (DIMEC), University of Bologna, 40126 Bologna, Italy; mirella.falconi@unibo.it

**Keywords:** cellular senescence, alveolospheres, inflammation, idiopathic pulmonary fibrosis, tissue remodeling related to epithelial–mesenchymal transition

## Abstract

Idiopathic pulmonary fibrosis (IPF) is one of the most severe forms of idiopathic interstitial pneumonia. Increasing evidence indicates that the gradual accumulation of senescent fibroblasts and alveolar epithelial cells contributes significantly to IPF pathogenesis, suggesting senescence as a potentially targetable process. Recurrent injury to the alveolar epithelium promotes senescence in epithelial cells, impairing their regenerative capacity and thereby predisposing the tissue to fibrotic degeneration. Although epithelial senescence is strongly implicated in the initiation and progression of lung fibrosis, the mechanisms through which it drives IPF remain challenging, partially due to the lack of physiologically relevant in vitro models capable of recapitulating lung architecture under both normal and pathological conditions. The objective of the present study was to develop a reproducible alveolosphere model in healthy and senescent conditions as a preliminary approach to investigate epithelial features that may be relevant to aspects of the IPF microenvironment. An alveolosphere system was generated by culturing alveolar epithelial cells with or without basement membrane components in combination with alveolar/epithelial optimized medium. Cultures were maintained for 3, 6, and 8 days, and cell viability together with morphological assessment confirmed the absence of cytotoxicity. The expression of keratin 8/18 and AQP5 was consistent with the maintenance of epithelial and alveolar-associated features. Cellular senescence was induced by exposing alveolospheres to doxorubicin for 24 h. Subsequent analyses of viability, along with the expression of senescent and pro-fibrotic markers, inflammatory mediators, and tissue remodeling factors, such as MMPs, were carried out in senescent 3D structures. The results demonstrated robust cell viability at all time points, supported by morphological observations. Marker expression suggested preservation of key epithelial characteristics, while senescence-inducing conditions were associated with an increase in senescence-associated, pro-fibrotic, inflammatory, and matrix-modulating markers. Collectively, these findings describe the preliminary establishment of a cost-effective and reproducible alveolosphere platform that may represent a useful starting point for studying epithelial senescence and its potential association with pro-fibrotic signaling relevant to aspects of IPF pathogenesis. Furthermore, this model may provide a basis for the preliminary evaluation of senotherapeutic compounds aimed at delaying or preventing the onset of cellular senescence.

## 1. Introduction

Idiopathic pulmonary fibrosis (IPF) is a fibrotic interstitial lung disease (fILD) characterized by progressive scarring of the lung parenchyma and a consequent decline in lung function [1,2]. Following repeated micro-injuries to the alveolar epithelium, alveolar epithelial type I cells (AEC1) undergo cell death and are replaced by alveolar epithelial type II cells (AEC2), which subsequently transdifferentiate into AEC1 cells [3,4]. Genetic predisposition, together with environmental triggers and comorbidities commonly associated with aging, can impair the reparative capacity of the alveolar epithelium, thereby promoting the onset of pathological conditions [5,6]. A substantial loss of AEC2 cells—due to apoptosis and/or cellular senescence—may facilitate the transition of epithelial cells toward a mesenchymal phenotype through epithelial-to-mesenchymal transition (EMT) [7,8]. EMT is a hallmark of IPF and is characterized by the accumulation of aberrantly activated myofibroblasts that secrete excessive amounts of extracellular matrix (ECM) components, including collagens I, III, and VI, as well as fibronectin. Excessive ECM deposition increases lung stiffness and density, alters lung biomechanics, and ultimately compromises the respiratory cycle [7,8]. Current antifibrotic therapies modestly slow IPF progression but do not reverse the disease, while lung transplantation remains the intervention associated with the greatest survival benefit in severe cases [9,10]. Therefore, the development of more effective therapeutic strategies to counteract IPF onset and progression is urgently needed.

Growing evidence highlights the pivotal contribution of cellular senescence to IPF pathogenesis [11,12,13,14]. Within the fibrotic microenvironment, senescent AECs and fibroblasts accumulate and release a senescence-associated secretory phenotype (SASP), a pro-inflammatory and pro-fibrotic milieu that drives immune cell recruitment and excessive ECM deposition, ultimately promoting progressive architectural remodeling of the lung [11]. Senescent AEC2 cells exhibit impaired proliferative and regenerative capacity, sustain injury-related signaling, and indirectly potentiate fibroblast activation. SASP components include stroma-remodeling cytokines such as IL-6, TGF-β, and several matrix metalloproteinases capable of inducing myofibroblast activation and scar formation [14].

Cellular senescence in IPF may be triggered by oxidative stress, DNA damage induced by genetic or environmental factors, telomere attrition, mitochondrial dysfunction, oncogene activation, and ionizing radiation. Under these conditions, senescent AEC2 cells undergo irreversible cell-cycle arrest through activation of the p53-p21^Waf1/Cip1^ and p^16INK4a^–retinoblastoma (Rb) pathways, ultimately failing to sustain proper lung regeneration following micro-injuries. Single-cell RNA-sequencing studies reveal a significant enrichment of senescent AEC2 cells in the lung tissues of IPF patients compared with healthy controls, with abnormal activation of senescence-related pathways such as oxidative stress and p53 signaling [13]. Nonetheless, the mechanisms through which cellular senescence promotes IPF progression remain incompletely understood, and further studies are needed to elucidate the molecular basis of senescent AEC2-driven fibrogenesis and to support the development of senescence-targeted therapeutic approaches.

One major limitation in the development of strategies to reduce AEC2 senescence and IPF progression is the lack of physiologically relevant in vitro models that allow reliable testing of potential therapeutics. The lung, particularly the distal alveolar region, is characterized by complex architecture and multiple interacting cell types, which are difficult to recapitulate in vitro. The development of 3D lung models that replicate the cellular heterogeneity, geometry, and biomechanics of the distal lung microenvironment enables more accurate investigation of cellular interactions under physiological and pathological conditions [15]. Although some studies have demonstrated the feasibility of lung organoids and spheroids [16], only a few have explored their relevance to cellular senescence and IPF.

The objective of this study is to establish a reproducible 3D model of the alveolosphere under both healthy and senescent conditions as a preliminary experimental system to investigate epithelial features potentially relevant to aspects of the in vivo IPF environment. To this aim, AEC2 cells were cultured in spheroid conditions, with or without basement membrane components (GeltrexTM), in control and epithelial/alveolar optimized medium. Transmission electron microscopy (TEM) showed features suggestive of alveolar-like organization, while the expression of keratin 8/18 and AQP5 proteins was consistent with the presence of epithelial and alveolar-associated characteristics. Cellular senescence was induced through doxorubicin-mediated DNA damage. The expression of senescent, pro-fibrotic, inflammatory mediators and tissue remodeling markers was assessed.

Overall, the results demonstrate a cost-effective and reproducible preliminary alveolosphere model that may be useful for investigating lung epithelial senescence and its potential association with pro-fibrotic processes relevant to aspects of IPF pathogenesis. Moreover, this model may provide a basis for future studies aimed at evaluating potential senotherapeutic agents aimed at delaying or preventing the onset of cellular senescence.

## 2. Results

### 2.1. In Vitro Alveolosphere Model

**Figure 1:**
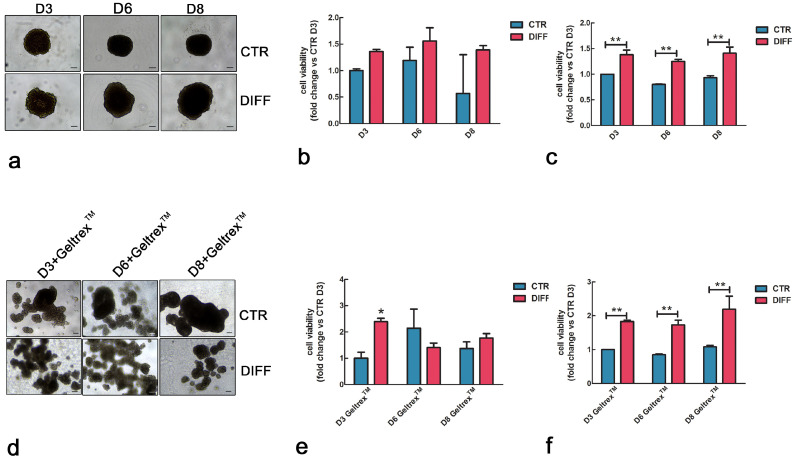
(**a**) Representative light microscopy images showing alveolospheres in vitro cultured in conditions free of the basement membrane extract Geltrex™, in control and commercial epithelial/alveolar differentiation medium. Alveolospheres consisted of human lung A549 cells cultured with control (CTR) and differentiation medium (DIFF) for 3 (D3), 6 (D6), and 8 days (D8) (bar: 50 μm). (**b**) MTT cell viability assay on alveolospheres in vitro cultured with differentiation medium and lacking the basement membrane extract Geltrex™. The data are expressed as fold changes vs. the control sample after 3 days of culture (CTR D3) ± SD. No statistically significant differences were observed. (**c**) Resazurin cell viability assay on alveolospheres in vitro cultured with differentiation medium and lacking the basement membrane extract Geltrex™. The data are expressed as fold changes vs. the control sample after 3 days of culture (CTR D3) ± SD. **represents a significant difference compared to each CTR; *p* < 0.01. (**d**) Representative light microscopy images showing alveolospheres in vitro cultured with epithelial/alveolar differentiation medium and Geltrex™. Alveolospheres consisted of human lung A549 cells cultured with control (CTR) and differentiation medium (DIFF) for 3 (D3), 6 (D6), and 8 days (D8) (bar: 100 μm). (**e**) MTT cell viability assay on alveolospheres in vitro cultured with differentiation medium and Geltrex™. The data are expressed as fold changes vs. the control sample after 3 days of culture (CTR D3) ± SD. * represents a significant difference compared to CTR D3; *p* < 0.05. (**f**) Resazurin cell viability assay on alveolospheres in vitro cultured with differentiation medium and the basement membrane extract Geltrex™. The data are expressed as fold changes vs. the control sample after 3 days of culture (CTR D3) ± SD; ** represents a significant difference compared to each CTR; *p* < 0.01.

To establish a reproducible alveolosphere model, A549 cells were seeded in low-attachment multi-well plates and cultured with commercial epithelial/alveolar optimized medium for 3, 6, and 8 days. Under both control and differentiation conditions, a single spheroid was generally observed in each well and increased in size in a time-dependent manner (Figure 1a). Cell viability assessed by MTT (Figure 1b) and resazurin-based assays (Figure 1c) revealed high viability levels in both conditions, with a 1.5-fold increase observed after 6 and 8 days in differentiation medium compared with the D3 control sample (Figure 1b,c).

We compared these results with A549 cells cultured under the same conditions as previously described, but in the presence of the basement membrane extract Geltrex™. In samples induced to differentiation, the formation of multiple small and fragmented spheroids was observed, whereas control samples developed irregular yet compact spheroids (Figure 1d). MTT and resazurin assays indicated maintained levels of cell viability in both control and differentiated cultures, with a 2-fold increase observed after 3 and 6–8 days of differentiation (Figure 1e,f).

### 2.2. Expression of the Epithelial/Alveolar Markers Keratin 8/18 and AQP5

To identify which spheroid culture condition more closely supports alveolar-associated features and promotes the expression of the epithelial/alveolar markers keratin 8/18 and AQP5, respectively, Western blot analyses were performed.

**Figure 2:**
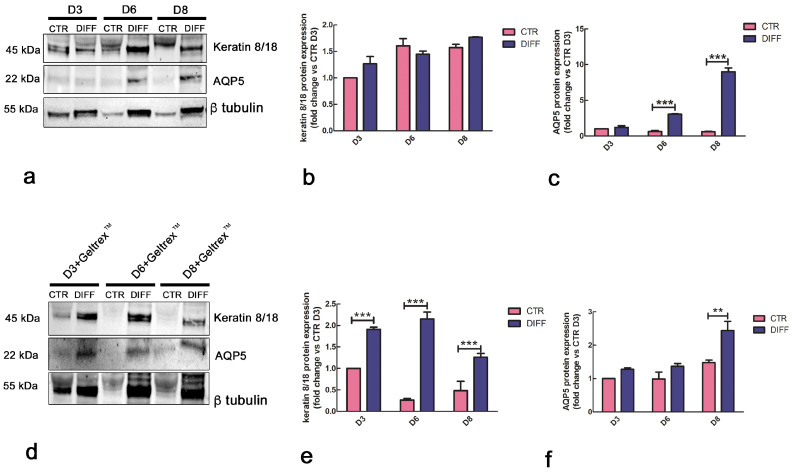
(**a**) Representative Western blot images showing Keratin 8/18 and AQP5 protein expression in alveolospheres in vitro cultured with epithelial/alveolar optimized medium and lacking Geltrex™. Alveolospheres consisted of human lung A549 cells cultured with control (CTR) and differentiation medium (DIFF) for 3 (D3), 6 (D6), and 8 days (D8). Densitometry analysis of the protein Keratin 8/18 (**b**) and AQP5 (**c**) bands. The amounts of the specific protein were normalized to the intensity of β-tubulin, and the relative quantification was expressed as relative value vs. CTR D3 ± SD. Statistical analysis showed a significant difference compared to the respective CTR sample. *** *p* < 0.001; (**d**) representative Western blot images showing Keratin 8/18 and AQP5 expression in alveolospheres in vitro cultured with the basement membrane extract Geltrex™ and with epithelial/alveolar optimized medium. Alveolospheres consisted of human lung A549 cells cultured with control (CTR) and differentiation medium (DIFF) for 3 (D3), 6 (D6), and 8 days (D8). Densitometry analysis of the protein Keratin 8/18 (**e**) and AQP5 (**f**) bands. The amounts of the specific protein were normalized to the intensity of β-tubulin, and the relative quantification was expressed as relative value vs. CTR D3 ± SD. Statistical analysis showed a significant difference compared to the respective CTR sample. ** *p* < 0.01; *** *p* < 0.001.

In spheroids cultured in the absence of the basement membrane extract Geltrex™, keratin 8/18 protein was detectable in all samples at every time point examined (Figure 2a). Densitometric quantification revealed a positive, though not statistically significant, trend toward increased keratin 8/18 expression in differentiated spheroids relative to their respective controls (Figure 2b). AQP5 expression was minimal in both control and differentiated spheroids at day 3 (Figure 2a). However, spheroids differentiated for 6 and 8 days exhibited a marked, time-dependent increase in AQP5 signal, with a statistically significant approximately 9-fold upregulation observed at day 8 (Figure 2c).

In spheroids cultured in the presence of Geltrex™, keratin 8/18 expression was significantly increased in all differentiated samples compared with their respective controls (Figure 2d,e), with densitometric analysis showing approximately a 2-fold upregulation at days 3 and 6 and a comparatively smaller, though still significant, increase at day 8 (Figure 2e). AQP5 protein, while present at overall low levels, was consistently detectable in all differentiated Geltrex™ spheroids, whereas control samples showed an almost undetectable signal at days 3 and 6 (Figure 2d). Densitometric analysis confirmed a significant, approximately 2-fold increase in AQP5 expression in differentiated alveolospheres relative to controls at day 8 (Figure 2f).

Taken together, these results suggest that, in the absence of Geltrex™, prolonged differentiation (6–8 days) was required to induce higher AQP5 expression, whereas Geltrex™ spheroids displayed earlier induction of keratin 8/18, though with a more modest and later-onset increase in AQP5.

### 2.3. Ultrastructural Morphology of Alveolospheres

TEM was performed to investigate the ultrastructural organization of A549 spheroids cultured with or without the basement membrane extract Geltrex™ and stimulated toward alveolar differentiation.

**Figure 3:**
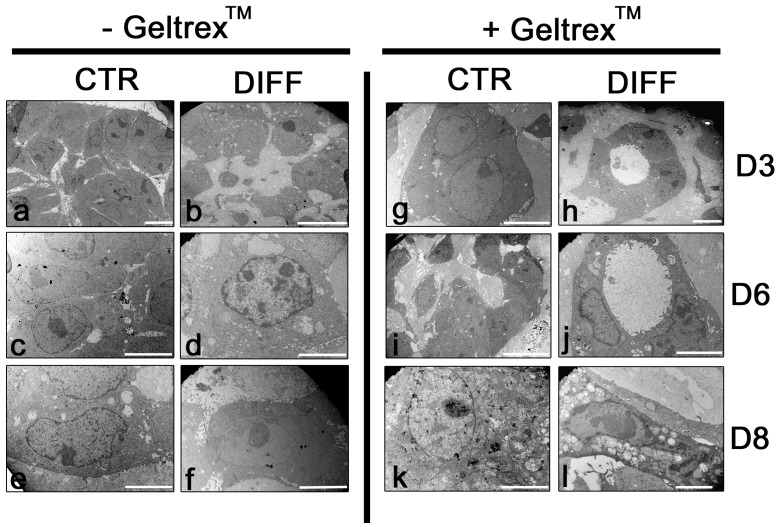
Representative TEM images showing alveolospheres in vitro cultured in condition-free (−Geltrex™) or with the basement membrane extract Geltrex™ (+Geltrex™), and they were cultured in control (CTR) and epithelial/alveolar optimized medium (DIFF) for 3 (D3), 6 (D6), and 8 days (D8). (**a**) Control spheroids cultured for 3 days lacking Geltrex™ (bar: 20 μm); (**b**) differentiated spheroids cultured for 3 days lacking Geltrex™ (bar: 20 μm); (**c**) control spheroids cultured for 6 days lacking Geltrex™ (bar: 5 μm); (**d**) differentiated spheroids cultured for 6 days lacking Geltrex™ (bar: 5 μm); (**e**) control spheroids cultured for 8 days lacking Geltrex™ (bar: 5 μm); (**f**) differentiated spheroids cultured for 8 days lacking Geltrex™ (bar: 20 μm); (**g**) control spheroids cultured for 3 days with Geltrex™ (bar: 5 μm); (**h**) differentiated spheroids cultured for 3 days with Geltrex™ (bar: 10 μm); (**i**) control spheroids cultured for 6 days with Geltrex™ (bar: 20 μm); (**j**) differentiated spheroids cultured for 6 days with Geltrex™ (bar: 5 μm); (**k**) control spheroids cultured for 8 days with Geltrex™ (bar: 5 μm); (**l**) differentiated spheroids cultured for 8 days with Geltrex™ (bar: 5 μm).

Spheroids generated without Geltrex™ exhibited a comparable ultrastructural morphology in both control and differentiated conditions (Figure 3). In all samples, at 3, 6, and 8 days, cells formed compact clusters tightly interconnected, consistent with the solid and homogeneous architecture observed by light microscopy (Figure 3a–f). At higher magnification, cells displayed a preserved polygonal morphology, a centrally located nucleus, and abundant mitochondria in the perinuclear region (Figure 3d–f). No ultrastructural hallmarks associated with alveolar differentiation—such as lumen formation or epithelial thinning—were detected at any time point.

In contrast, spheroids cultured in the presence of Geltrex™ and stimulated toward alveolar differentiation displayed different ultrastructural features (Figure 3g–l). Structures consisting of a monolayer of cells arranged in circular formations resembling alveolar-like structures were observed as early as day 3 (Figure 3h). These formations persisted and became more defined at day 6, with cells adopting a more elongated morphology suggestive of epithelial rearrangement (Figure 3j). Control spheroids cultured with Geltrex™ maintained a compact structure composed of small polygonal cells and showed no evidence of ring-like organization (Figure 3g,k). At day 8, alveolar-like structures remained detectable in differentiated samples; however, several signs of cellular distress were evident (Figure 3l). These included chromatin condensation and mitochondrial vacuolization, morphological alterations typically associated with apoptotic processes. Based on these observations, the Geltrex™ condition was considered the most suitable among those tested and was therefore used for subsequent analyses.

### 2.4. Induction of Cellular Senescence in the Alveolosphere Model

To establish senescence-inducing conditions in the alveolosphere-based system and investigate cellular responses potentially relevant to fibrotic disease, alveolospheres generated in the presence of the basement membrane extract Geltrex™ were treated with doxorubicin, a DNA-intercalating agent that blocks cell-cycle progression.

**Figure 4:**
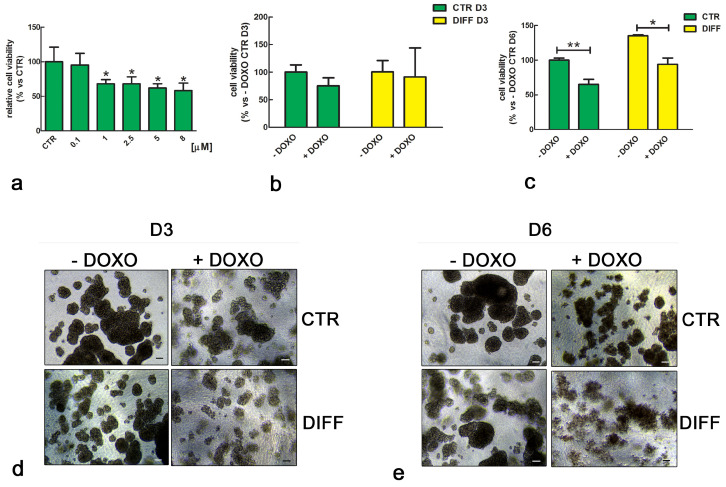
(**a**) Cell viability assay on A549 cultured in a monolayer exposed to gradual concentrations of doxorubicin for 24 h. Data are expressed in percentages, and they are represented as relative value ± SD vs. CTR sample consisting of A549 cells lacking doxorubicin treatment. Statistical analysis demonstrated a significant difference compared to CTR, * *p* < 0.05; (**b**) cell viability assay on alveolosphere differentiated for 3 days (D3) and subsequently exposed to 2.5 μM of doxorubicin (DOXO) for 24 h. Data are expressed in percentages, and they are represented as relative value ± SD vs. CTR D3 sample (CTR D3–DOXO). No statistical difference was detected; (**c**) cell viability assay on alveolosphere differentiated for 6 days (D6) and subsequently exposed to 2.5 μM of doxorubicin (DOXO) for 24 h. Data are expressed in percentages, and they are represented as relative values ± SD vs. CTR D6 sample (CTR D6–DOXO). Statistical analysis demonstrated a significant difference compared to each untreated sample. * *p* < 0.05; ** *p* < 0.01. (**d**) Representative light microscopy images showing alveolospheres in vitro cultured with Geltrex™, differentiated (DIFF) for 3 days (D3) and subsequently exposed to 2.5 μM of doxorubicin (+DOXO) for 24 h. Control samples consisted of A549 cells in vitro cultured with Geltrex™ for 3 days and lacking differentiation medium (CTR) and/or DOXO (−DOXO) (bar: 100 μm). (**e**) Representative light microscopy images showing alveolospheres in vitro cultured with Geltrex™, differentiated (DIFF) for 6 days (D6) and subsequently exposed to 2.5 μM of doxorubicin (+DOXO) for 24 h. Control samples consisted of A549 cells in vitro cultured with Geltrex™ for 6 days and lacking differentiation medium (CTR) and/or DOXO (−DOXO) (bar: 100 μm).

As a preliminary step, doxorubicin cytotoxicity was assessed in A549 monolayers by MTT assay. A progressive, concentration-dependent decrease in cell viability was observed, reaching approximately 52% at 8 μM (Figure 4a). Aiming to induce mild stress while minimizing overt cytotoxicity, we selected 2.5 μM doxorubicin—corresponding to ~70% viability—for subsequent experiments (Figure 4a).

Alveolospheres differentiated for 3 or 6 days were then exposed to 2.5 μM doxorubicin for 24 h. At day 3, MTT analysis revealed a modest, non-significant reduction in viability following treatment (Figure 4b). Conversely, at day 6, doxorubicin exposure resulted in a modest but significant decrease in cell viability (Figure 4c) compared to each untreated sample.

Light microscopy supported these findings. At day 3, both untreated and doxorubicin-treated samples consisted of small, compact spheroid clusters with no appreciable morphological alterations (Figure 4d). At day 6, untreated cultures displayed numerous small yet intact spheroids (Figure 4e), whereas doxorubicin-treated samples, although similar in number and size, appeared fragmented, consistent with early signs of cellular distress (Figure 4e).

### 2.5. Expression of Cellular Senescence Markers in the Alveolosphere Model

To verify the induction of cell cycle arrest, a hallmark of the senescent phenotype, we analyzed the expression of the markers p21^Waf1/Cip1^ and p16^INK4a^.

**Figure 5:**
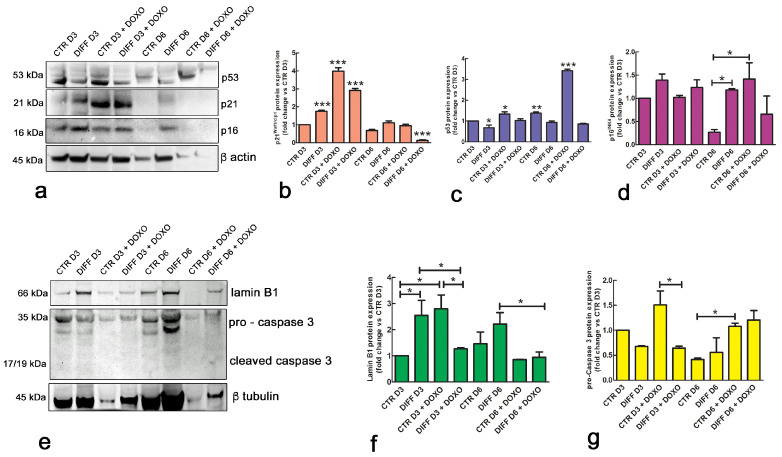
(**a**) Representative Western blot images showing p21^Waf1/Cip1^, p53, and p16^INK4a^ protein expression in control (CTR) and differentiated alveolospheres (DIFF) cultured in vitro for 3 (D3) and 6 (D6) days. Some samples were treated with 2.5 μM doxorubicin for 24 h (+DOXO). β-actin represents the loading control. Densitometry analysis of the p21^Waf1/Cip1^ (**b**), p53 (**c**), and p16^INK4a^ (**d**) protein bands. The relative amounts of the protein expression were normalized to the intensity of β-actin and represented as a fold increase relative to CTR D3± SD. * *p* < 0.05; ** *p* < 0.01; *** *p* < 0.001; (**e**) representative Western blot images showing lamin B1, pro-caspase 3, and cleaved caspase 3 protein expression in control (CRT) and differentiated alveolospheres (DIFF) cultured in vitro for 3 (D3) and 6 (D6) days. Some samples were treated with 2.5 μM doxorubicin for 24 h (+DOXO). β-tubulin represents the loading control. Densitometry analysis of lamin B1 (**f**) and pro-caspase 3 (**g**) protein bands. Relative protein expression was normalized to the β-tubulin intensity, and it is represented as a fold increase relative to CTR D3 ± SD; * *p* < 0.05.

Results showed an increase in p21^Waf1/Cip1^ protein levels in control and differentiated samples cultured for 3 days and exposed to doxorubicin (Figure 5a). Densitometric analysis confirmed a 4-fold and a 3-fold upregulation of the protein, respectively, in control and differentiated samples cultured for 3 days and then exposed to doxorubicin (Figure 5b). Conversely, this effect was less pronounced in alveolospheres differentiated for 6 days and then exposed to doxorubicin treatment (Figure 5a,b).

Given its key role in the DNA damage response (DDR) and its correlation with p21^Waf1/Cip1^, we subsequently assayed p53 expression. The protein was detected across all experimental conditions, with a modest trend toward upregulation in most doxorubicin-treated samples. Notably, a substantial increase was observed in control alveolospheres treated with doxorubicin for 6 days, which exhibited a 3-fold higher expression compared to the D3 control sample (Figure 5a,c).

In contrast, expression of p16, a marker typically associated with maintenance of the senescent phenotype, did not show a clear DOXO-dependent induction at either time point (Figure 5a,d), suggesting that the experimental conditions examined may primarily reflect an early response involving the p53/p21 pathway rather than a fully established p16/Rb-dependent maintenance phase of senescence.

To further investigate the presence of senescence-associated alterations, additional markers of nuclear integrity and apoptosis were evaluated (Figure 5e). Lamin B1, whose downregulation is a hallmark of senescence, was reduced in DOXO-treated differentiated spheroids at both day 3 and day 6 (Figure 5e,f) compared with untreated differentiated controls, supporting a senescence-associated remodeling of the nuclear lamina that persisted beyond the early p21-dependent response.

Regarding the activation of apoptosis following doxorubicin exposure, no cleaved (active) fragment of caspase 3 was detected in any condition, with only the full-length pro-caspase 3 form (~35 kDa) observed throughout (Figure 5e). Densitometric quantification of pro-caspase 3 levels showed overall stable expression across conditions, with only isolated, modest significant differences between specific sample pairs (Figure 5g) and no evidence of a consistent depletion of the precursor pool following doxorubicin treatment. Together with the absence of detectable cleaved fragments, these data indicate that doxorubicin treatment, at the dose and exposure time used, did not trigger overt caspase 3-mediated apoptosis.

Taken together, these results suggest that DOXO exposure induces a senescence-associated phenotype in A549 alveolospheres characterized by early activation of the p53/p21 axis at day 3, followed by progressive loss of Lamin B1 at both day 3 and day 6, in the absence of caspase 3-mediated apoptotic activation. The lack of p16 induction further suggests that, under these experimental conditions, the response may predominantly involve early p21-associated senescence-related mechanisms rather than a transition to a p16/Rb-mediated maintenance state.

### 2.6. Investigation of SASP Components

Senescent cells are characterized by the secretion of a complex array of pro-inflammatory factors collectively known as SASP. Accordingly, the expression of key SASP components, including IL-1β, IL-8, and TNF-α, as well as the release of IL-6 into the culture medium, was investigated in alveolospheres cultured in Geltrex™ and exposed to doxorubicin.

**Figure 6:**
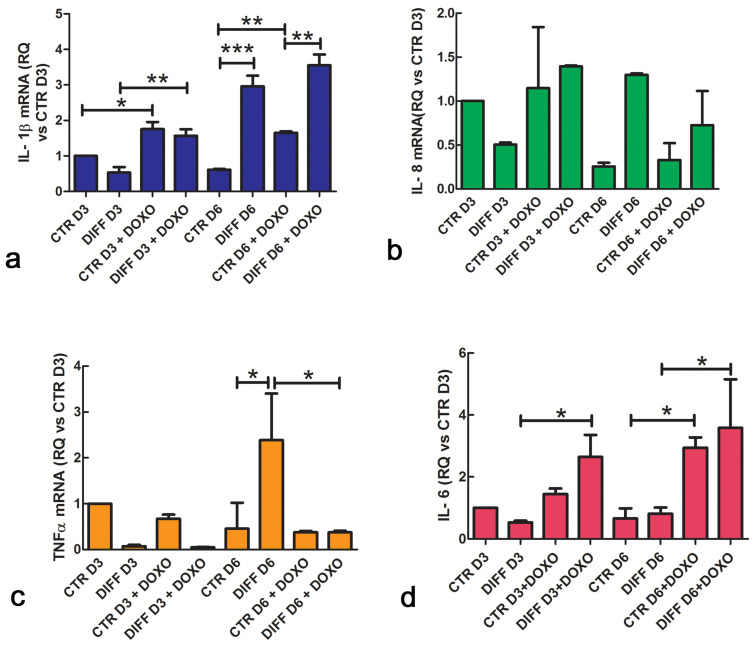
RT-qPCR quantification of IL-1β (**a**), IL-8 (**b**), and TNF-α (**c**) mRNA expression in control (CTR) and differentiated (DIFF) alveolospheres cultured for 3 (D3) and 6 (D6) days in epithelial/alveolar optimized medium and Geltrex™ in the presence or absence of doxorubicin (DOXO). Data are expressed as fold change relative to the control group (CTR D3). (**d**) IL-6 protein levels in the culture medium, determined by immunoassay in control (CTR) differentiated (DIFF) alveolospheres cultured for 3 (D3) and 6 (D6) days and treated with doxorubicin (DOXO). Data are expressed as fold change relative to CTR D3. All values represent the mean ± SD of three independent experiments. * *p* < 0.05; ** *p* < 0.01; *** *p* < 0.001.

RT-qPCR analysis revealed a significant upregulation of IL-1β mRNA expression in doxorubicin-treated alveolospheres with a more pronounced effect in differentiated samples, reaching a maximum induction of approximately 3.5-fold in samples differentiated for 6 days and subsequently exposed to doxorubicin (Figure 6a). In contrast, IL-8 mRNA levels did not show any significant variation across the experimental conditions, irrespective of differentiation status or doxorubicin treatment (Figure 6b).

Similarly, TNF-α expression did not show a doxorubicin-dependent modulation in all analyzed samples when compared with control alveolospheres (Figure 6c).

Conversely, analysis of IL-6 protein release in the culture medium revealed a progressive increase in cytokine secretion in differentiated alveolospheres and in samples exposed to doxorubicin (Figure 6d). Notably, the highest IL-6 levels were detected in differentiated alveolospheres treated with doxorubicin, reaching up to a 4-fold increase relative to control samples (Figure 6d).

Collectively, these findings are consistent with the modulation of selected SASP-associated factors following doxorubicin treatment, with IL-1β and IL-6 emerging as the most prominently induced pro-inflammatory mediators under senescence-inducing conditions.

### 2.7. Expression of Pro-Fibrotic Mediators and Matrix Remodeling Enzymes in Doxorubicin-Treated Alveolospheres

To further characterize the molecular changes associated with senescence-inducing conditions, the expression of the pro-fibrotic mediator TGF-β1 and of matrix remodeling enzymes MMP1, MMP2, and MMP9 was analyzed by RT-qPCR in differentiated alveolospheres cultured in Geltrex™ and exposed to doxorubicin.

**Figure 7:**
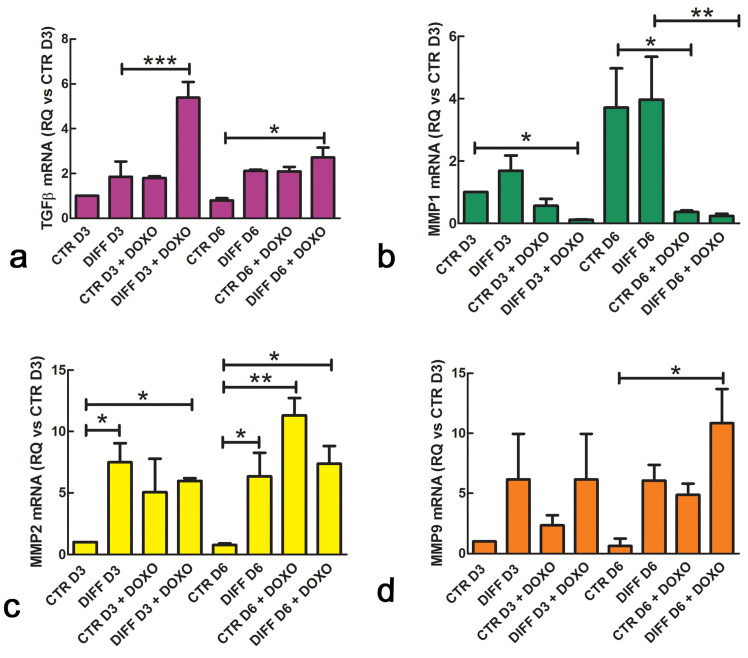
RT-qPCR analysis of TGF-β1 (**a**), MMP1 (**b**), MMP2 (**c**), and MMP9 (**d**) mRNA expression in control (CTR) and differentiated (DIFF) alveolospheres cultured for 3 (D3) and 6 (D6) days in epithelial/alveolar optimized medium and Geltrex™, in the presence or absence of doxorubicin (DOXO). Gene expression levels are expressed as fold change relative to the control group (CTR D3). All data represent the mean ± SD of three independent experiments. * *p* < 0.05; ** *p* < 0.01; *** *p* < 0.001.

TGF-β1 mRNA levels were markedly increased in alveolospheres differentiated for 3 days and treated with doxorubicin, reaching an induction of approximately 6-fold compared with control samples (Figure 7a). Although still statistically significant, TGF-β1 expression was reduced in alveolospheres differentiated for 6 days and exposed to doxorubicin (Figure 7a), suggesting that the effect was more evident at the earlier time point.

Analysis of MMP1 expression revealed a different regulation depending on the experimental condition. Control and differentiated alveolospheres for 3 and 6 days were associated with a moderate increase in MMP1 expression, whereas doxorubicin treatment resulted in a marked downregulation of MMP1 levels at both 3 and 6 days of culture (Figure 7b), indicating an association between doxorubicin exposure and reduced MMP1 expression under the conditions examined.

In contrast, MMP2 expression was strongly induced in the model. A moderate but significant increase in MMP2 levels (up to approximately 5-fold) was observed in differentiated alveolospheres and/or in samples exposed to doxorubicin (Figure 7c). Notably, the combination of prolonged differentiation (6 days) and doxorubicin treatment resulted in a robust upregulation of MMP2 expression, reaching nearly a 10-fold increase compared with control alveolospheres (Figure 7c).

A similar trend was observed for MMP9. While the different experimental conditions showed an upward trend in MMP9 expression, a statistically significant increase—approaching a 10-fold induction—was detected exclusively in alveolospheres differentiated for 6 days and treated with doxorubicin (Figure 7d). Other conditions exhibited variable, non-significant changes relative to the control sample.

Collectively, these results suggest that doxorubicin-treated alveolospheres develop molecular alterations involving selected pro-fibrotic and matrix-remodeling mediators, characterized by increased TGF-β1, MMP2, and MMP9 expression together with reduced MMP1 levels. Further studies will be required to determine the extent to which these changes reflect processes occurring in the IPF microenvironment.

## 3. Discussion

In vitro modeling of the lung parenchyma remains highly challenging due to the remarkable cellular complexity of the pulmonary microenvironment, which comprises not only airway epithelial cells but also AECI and AECII cells, macrophages, mesenchymal stromal cells, endothelial cells, pericytes, and multiple immune cell populations. All these cell types play distinct and essential roles in maintaining lung structure and function [17].

Dysregulation or dysfunction of one or more of these cellular populations is implicated in the pathogenesis of several respiratory diseases. Consequently, the development of a single, comprehensive 3D pulmonary model capable of faithfully reproducing multiple lung pathologies is technically demanding, economically costly, and often unsuitable for routine in vitro studies.

We focus our research on IPF, a fibrotic interstitial lung disease characterized by progressive scarring of the lung parenchyma, whose pathogenesis has not yet been fully elucidated [1]. Following repeated alveolar injury, chronic inflammation and pro-fibrotic signaling pathways are activated, promoting EMT and the emergence of myofibroblasts. These cells are responsible for excessive extracellular matrix deposition, ultimately leading to increased lung tissue stiffness and fibrosis [7].

Over the past decade, increasing attention has been directed toward alveolar cellular senescence as a key driver of chronic pulmonary inflammation following epithelial damage. Senescent AECs and fibroblasts accumulate within the lung parenchyma and secrete SASP components. These factors promote persistent inflammation, defective autophagic flux, impaired proteostasis, and mitochondrial dysfunction, thereby amplifying cellular stress signaling and contributing to IPF development and progression [12].

Although several in vitro 3D models have been proposed to simulate physiological lung architecture and respiratory diseases [17,18], only a limited number specifically address IPF. Moreover, most of these models rely on complex biomaterials in combination with induced pluripotent stem cells, resulting in systems that are costly, technically demanding, and often poorly reproducible [15]. Therefore, the aim of this study was to develop a cost-effective and reproducible preliminary alveolosphere-based system under senescence-inducing conditions, suitable for investigating selected epithelial features potentially relevant to aspects of the IPF microenvironment. Such a system may provide a useful experimental framework for studying cellular responses associated with senescence and for the preliminary evaluation of candidate senotherapeutic compounds.

Since alveolar senescence is mainly associated with damage to alveolar AEC2, the A549 lung cell line was used to establish an alveolosphere model. A549 cells have been extensively characterized in the literature as type II pneumocyte-like cells at the ultrastructural morphology, biochemical, and molecular level [19]. Although A549 cells are derived from a human pulmonary adenocarcinoma, this cell line is genetically stable, making the 3D model highly reproducible and cost-effective. Moreover, the use of A549 cells avoids genetic reprogramming procedures required for induced pluripotent stem cells derived from adult primary AEC2 [20]. At the same time, this approach allows the use of a cell population that already displays an AEC2-like phenotype, providing appropriate baseline conditions for the induction and study of cellular senescence.

Although A549 cells offer important practical advantages for establishing a reproducible and cost-effective alveolosphere system, we believe that primary human AEC2 cells represent a more physiologically relevant cellular source for modeling the alveolar epithelium. Preliminary attempts to generate alveolospheres using primary human alveolar epithelial cells were performed; however, reproducible expansion and long-term culture could not be consistently achieved under the experimental conditions tested. Therefore, A549 cells were selected for the present study. Nevertheless, the use of primary human AEC2 cells remains a major objective for future developments of the model.

To establish a 3D alveolosphere model, A549 cells were cultured in vitro either in the absence or in the presence of a basement membrane component (Geltrex™), in combination with commercial epithelial/alveolar-optimized media to promote differentiation toward an AEC1-like phenotype. Under both culture conditions, no significant reduction in cell viability was observed after 8 days in either the control or differentiated environment. However, light microscopy analysis revealed markedly different 3D morphologies. In the absence of Geltrex™, cells formed a single, compact, and well-defined sphere, whereas cultures grown in the presence of Geltrex™ exhibited a more irregular, fragmented, and less organized structure, surrounded by several ultrasmall spheroids. TEM ultrastructural analysis showed that ultrasmall spheroids differentiated for 3 days displayed a monolayer organization of cells encircling a central lumen. After 6 days of differentiation, cells remained organized as a circular monolayer structure, exhibiting a more elongated cellular morphology. Overall, morphological analyses revealed the formation of structures displaying alveolar-like morphological features in samples differentiated for 3 and 6 days. To our knowledge, the specific morphological organization observed under these experimental conditions has been only limitedly described in comparable alveolar spheroid systems.

An additional aspect to consider during the establishment of the alveolosphere system was the potential emergence of fibroblast-like cells as a consequence of epithelial-to-mesenchymal transition (EMT) occurring within the A549 population. Such a phenomenon could potentially alter the cellular composition of the spheroids and influence the interpretation of the experimental findings. In the present study, TEM analysis did not reveal ultrastructural features typically associated with fibroblastic cells, supporting the predominance of an epithelial cell population within the analyzed structures. However, although TEM is a valuable tool for evaluating cellular ultrastructure, the examination of limited sample areas does not allow the definitive exclusion of fibroblast-like cells. Therefore, future studies incorporating fibroblast-specific molecular markers would provide a more comprehensive characterization of cell identity and further strengthen the characterization of the alveolosphere system.

To further assess the expression of epithelial and alveolar-associated features within the obtained alveolospheres, the expression of the epithelial and alveolar markers, respectively, keratin 8/18 and AQP5, was investigated. Keratin 8/18 is a general marker of epithelial cells; although it is not selective for AEC2, it is known to be expressed in alveolar epithelial cells, including AEC2-like populations [21]. In contrast, AQP5 is a well-established and selective marker of AEC1 cells [22]. Therefore, we hypothesized that the 3D culture conditions, in combination with differentiation medium, could promote the expression of markers associated with AEC1-like characteristics, thereby contributing to a more differentiated epithelial phenotype. Expression analyses further supported the observed morphological differences. In spheroids cultured without Geltrex™, keratin 8/18 was detected at all time points, whereas prolonged differentiation (6–8 days) was required to achieve a substantial increase in AQP5 expression. Conversely, in Geltrex™-containing cultures, differentiation was associated with an earlier induction of keratin 8/18 together with a progressive increase in AQP5 expression over time. Taken together, these findings suggest that the presence of a basement membrane component influences both the structural organization of A549-derived alveolospheres and the kinetics of epithelial/alveolar marker expression. Furthermore, considering the maintenance of alveolar-associated marker expression up to day 8 and the concomitant appearance of morphological alterations suggestive of cellular stress, this experimental system may be more suitable for short-term culture conditions.

To establish senescence-inducing conditions within the alveolosphere system and investigate cellular responses potentially relevant to fibrotic lung disease, alveolospheres were exposed to the DNA-intercalating agent doxorubicin for 24 h [23]. Activation of the senescence-related signaling pathways p53–p21^Waf1/Cip1^ and p^16INK4a^ [24,25] supported the induction of a senescent phenotype in alveolospheres differentiated for 3 days, whereas a time-dependent reduction in marker expression was observed at later stages. This senescence-associated phenotype was further supported by reduced Lamin B1 expression, consistent with senescence-associated nuclear remodeling, while the absence of cleaved caspase 3 across all conditions excluded overt apoptotic activation, favoring a senescence-associated rather than a purely cytotoxic response.

However, the analysis of canonical senescence markers alone is not sufficient to demonstrate the establishment of a senescent microenvironment [24,25]. For this reason, the expression of selected SASP components closely linked to senescence-driven inflammation was investigated [26,27]. The results revealed increased IL-1β expression and IL-6 release under selected experimental conditions, whereas IL-8 and TNF-α showed limited or no significant modulation, in agreement with previous studies describing the presence of senescent alveolar epithelial cells and their secretory profile in IPF [28,29]. These mediators are known to promote a pro-inflammatory microenvironment that supports EMT and ECM deposition [30,31,32]. Notably, a divergence within the SASP profile was observed for TNF-α, whose expression did not show significant changes in differentiated and/or doxorubicin-treated samples. In a senescent alveolar epithelial context, cytokines such as IL-1β, IL-6, and IL-8 are predominantly produced by epithelial cells and exert both pro-inflammatory and pro-fibrotic effects, partly through activation of the TGF-β1 signaling axis, a central pathway in IPF pathogenesis [33]. In contrast, TNF-α is primarily secreted by lung fibroblasts and macrophages and is more closely associated with immune-cell–driven inflammatory responses rather than epithelial senescence per se [34].

Finally, to assess whether the Geltrex™-based senescent alveolosphere model was able to produce mediators associated with fibrosis, we investigated the expression of TGF-β1, a central regulator of fibrotic signaling [33]. TGF-β1 expression was increased in all differentiated and/or doxorubicin-treated samples. Notably, in alveolospheres differentiated for 3 days and subsequently exposed to doxorubicin, TGF-β1 expression reached up to a six-fold increase. These findings are consistent with the literature highlighting the pivotal role of TGF-β1 in IPF pathogenesis, where this cytokine regulates the terminal differentiation of human lung fibroblasts and promotes extracellular matrix synthesis [33,35,36].

The inflammatory environment, characterizing IPF, together with EMT and myofibroblast formation, drives extensive tissue remodeling in which MMPs play a central role in modifying the lung parenchymal architecture. Accordingly, we investigated the expression of MMP1, MMP2, and MMP9, extracellular matrix-remodeling enzymes implicated in IPF pathogenesis [37].

Our results showed reduced MMP1 expression under doxorubicin-treated conditions, suggesting alterations in extracellular matrix regulatory pathways. In contrast, MMP2 and MMP9 expression was increased in differentiated alveolospheres and further enhanced following doxorubicin-induced senescence, although not always reaching statistical significance when compared with control samples. This expression pattern is consistent with an IPF-like scenario and supports the activation of EMT-associated remodeling pathways [8,38]. Elevated MMP2 and MMP9 levels are widely associated with basement membrane degradation and matrix remodeling processes that facilitate the initiation and progression of EMT [39].

Nevertheless, pro-fibrotic changes were assessed mainly at the transcriptional level, since the relatively short culture period of the current model is unlikely to support the deposition of a mature extracellular matrix. We acknowledge that histological and immunohistochemical analyses would provide a more comprehensive evaluation of matrix remodeling. Therefore, extending culture duration to enable extracellular matrix maturation and histological characterization represents an important objective for the future development of a more representative IPF-like microenvironment.

## 4. Materials and Methods

### 4.1. Monolayer Cell Culture and Alveolosphere Model

The human pulmonary adenocarcinoma cell line A549, widely recognized as AEC2 cells [19,40], was used for all experiments. Cells were used within passage 10 (P10) and were routinely tested for mycoplasma contamination; all cell culture reagents used throughout the study were commercially sourced.

A549 cells were maintained in Minimum Essential Medium (MEM) (Gibco, Thermo Fisher Scientific, Monza, Italy) supplemented with 10% fetal bovine serum (FBS) (Gibco, Thermo Fisher Scientific, Monza, Italy) and incubated in a humidified atmosphere containing 5% CO_2_ at 37 °C.

To generate alveolar spheroids, 5 × 10^3^ cells were seeded into low-attachment 96-well plates in MEM supplemented with 10% FBS and allowed to aggregate for 24 h. In a subset of samples, cells were mixed with Geltrex™ LDEV-Free Reduced Growth Factor Basement Membrane Matrix (Gibco, Thermo Fisher Scientific, Monza, Italy) following the manufacturer’s instructions to support extracellular matrix-dependent 3D structure formation. Geltrex™ was kept at 4 °C prior to seeding and subsequently maintained at 37 °C following seeding, consistent with standard handling recommendations. After 24 h, spheroids cultured with or without Geltrex™ were subjected to conditions promoting alveolar differentiation. Each experimental condition was analyzed using six biological replicates, and the entire study was independently repeated three times.

### 4.2. Alveolar Differentiation

After 24 h in MEM, spheroids generated with or without Geltrex™ were transferred to SABM™ Small Airway Epithelial Cell Growth Basal Medium (Lonza, Milan, Italy) supplemented with SAGM™ Small Airway Epithelial Cell Growth Medium Supplements and Growth Factors (Lonza, Milan, Italy) to promote differentiation toward an alveolar type I–like phenotype. Spheroids in both conditions (±Geltrex™) were cultured for 3, 6, and 8 days in a humidified atmosphere containing 5% CO_2_ at 37 °C. Control groups consisted of spheroids cultured in SABM™ medium lacking SAGM™ supplements.

At each designated time point, spheroids were imaged using an inverted light microscope (CKX53, Olympus Italia S.r.L., Segrate, Italy) and subsequently processed for downstream analyses.

### 4.3. Viability Assays

To assess cell viability in the spheroid models, a tetrazolium bromide (MTT) and resazurin-based assay (Presto Blue assay) were performed. Regarding the MTT assay, alveolospheres generated with or without Geltrex™ were seeded and maintained as previously described. At each experimental time point, the culture medium was replaced with fresh medium containing 5 µg/mL MTT (Sigma-Aldrich, St. Louis, MO, USA) and incubated for 3 h at 37 °C in a humidified atmosphere with 5% CO_2_. Following incubation, the resulting formazan crystals were solubilized by adding a 1:1 solution of dimethyl sulfoxide (DMSO) and isopropanol.

Optical density was measured using a GloMax^®^ Discover Microplate Reader (Promega Italia, Milan, Italy) at 578 nm, with a reference wavelength of 690 nm. Viability values were expressed as a percentage relative to control spheroids.

Regarding the Presto Blue assay (Invitrogen, Thermo Fisher Scientific, Monza, Italy), alveolospheres generated with or without Geltrex™ were seeded and maintained as previously described. At each experimental time point, 1/10th volume of the viability reagent was added to cells and incubated for 15 min at 37 °C. Fluorescence was measured using a GloMax^®^ Discover Microplate Reader (Promega Italia, Milan, Italy) with an excitation wavelength of 560 nm and an emission wavelength of 590 nm. Viability values were expressed as a percentage relative to control spheroids.

### 4.4. TEM Analysis

Alveolospheres cultured in both conditions (±Geltrex™) and incubated for 3, 6, and 8 days with SABM™ medium supplemented with SAGM™ growth factors were collected at the end of the incubation and fixed with 2.5% glutaraldehyde in 0.1 M cacodylate buffer for 2 h at 4 °C. They were subsequently post-fixed with 1% OsO_4_ in 0.1 M cacodylate buffer for 1 h at room temperature. After three washes with 0.15 M cacodylate buffer, the samples were dehydrated in an acetone series (25%, 50%, 70%, 90%, 100%) and embedded in Epon resin (Merk Life Science S.r.l., Milan, Italy).

Sections of 100 nm were collected on nickel grids, stained with uranyl acetate and lead citrate, and observed by Philips CM10 (FEI Company, Eindhoven, The Netherlands). Images were recorded by a Megaview III digital camera (FEI Company, Eindhoven, The Netherlands).

### 4.5. Cellular Senescence Induction

To determine the optimal concentration of doxorubicin required to induce cellular senescence, A549 cells were seeded in 24-well plates at a density of 8 × 10^3^ cells/well in MEM supplemented with 10% FBS. After 24 h of culture, the medium was replaced with fresh medium containing increasing concentrations of doxorubicin, and cells were incubated for an additional 24 h. At the end of the treatment, cell viability was assessed using the MTT assay as previously described.

Based on these results, 2.5 μM doxorubicin was selected as the most appropriate concentration to induce senescence in A549 monolayers. This concentration was subsequently applied to Geltrex™ alveolospheres cultured in SABM™ medium supplemented with SAGM™ growth factors, with parallel samples maintained in the absence of doxorubicin as controls. Spheroids were incubated for 3, 6, and 8 days at 37 °C in a humidified atmosphere containing 5% CO_2_. At each time point, cell viability was evaluated using the MTT assay as previously described.

### 4.6. Protein Extraction, SDS-PAGE, and Western Blot

Total protein was extracted using RIPA modified lysis buffer (Pierce, Thermo Fisher Scientific, Monza, Italy) supplemented with 25 μmol/L protease inhibitor cocktail (Pierce, Thermo Fisher Scientific, Monza, Italy) and 1 μL of β-mercapto-ethanol (Sigma-Aldrich, St. Louis, MO, USA). The amount of protein obtained from each sample was quantified by Bradford assay (Sigma-Aldrich, St. Louis, MO, USA), and 15 μg of total protein was separated by 4–12% sodium dodecyl sulfate polyacrylamide gel electrophoresis (SDS-PAGE), followed by transfer onto a nitrocellulose membrane (GE Healthcare, Amersham, UK). Subsequently, the membranes were incubated with 5% no fat milk (blocking reagent) to remove the nonspecific binding proteins, followed by incubation with the primary antibodies against anti-Keratin 8/18 antibody (BK4546T, Cell Signaling Technologies, Euroclone, Milan, Italy), anti-AQP5 antibody (BK59558S, Cell Signaling Technologies, Euroclone, Milan, Italy), anti-p21^Waf1/Cip1^ antibody (2947S, Cell Signaling Technologies, Euroclone, Milan, Italy), anti-p16^INK4a^ antibody (18769, Cell Signaling Technologies, Euroclone, Milan, Italy), anti p53 antibody (2524S Cell Signaling Technologies, Euroclone, Milan, Italy), anti-Lamin b1 antibody (12586 Cell Signaling Technologies, Euroclone, Milan, Italy), anti-caspase 3 antibody (9662 Cell Signaling Technologies, Euroclone, Milan, Italy), (anti-β-tubulin antibody (2146S Cell Signaling Technologies, Euroclone, Milan, Italy) and anti-β-actin antibody (3700S Cell Signaling Technologies, Euroclone, Milan, Italy). All the primary antibodies were diluted 1:1000 in blocking reagent at 4 °C overnight. After washing with TBS-tween 20 buffer, samples were incubated with HRP-linked anti-rabbit IgG secondary antibodies or HRP-linked anti-mouse IgG secondary antibodies, diluted 1:5000 in TBS-Tween 20 buffer (Sigma Aldrich, St. Louis, MO, USA). The antibody signal was visualized by the enhanced chemiluminescence system (Pierce, Thermo Fisher Scientific, Monza, Italy). Images were obtained by using the IBright Western Blot Imaging System. The densitometric analysis was performed using Image J software, version 1.53a (National Institutes of Health, Bethesda, MD, USA). The intensities of the specific protein bands were corrected for equal β-tubulin and β-actin signal, and they were expressed as relative values compared to the intensity of the respective control sample. Data showed the average of triplicates ± SD.

### 4.7. RNA Extraction and qRT-PCR

Total RNA was extracted from cell pellets using a PureLink™ RNA mini kit (Invitrogen, ThermoFisher, Monza, Italy) and quantified using a NanoDrop ND-1000 UV-Vis Spectrophotometer (ThermoFisher Scientific, Monza, Italy). The total RNA (100 ng) was reverse transcribed into first-strand cDNA using the SuperScript™ III One-Step RT-PCR System (Invitrogen, ThermoFisher Scientific, Monza, Italy). The expression of mRNA was analyzed by qRT-PCR using a 7500 Real-Time PCR instrument (Applied Biosystem, Life Technologies, Monza, Italy). For the analysis, the following TaqMan assays (Applied Biosystems, Life Technologies, Monza, Italy) were used: human IL-1β (Hs01555410_m1), IL-8 (Hs00174103_m1), TNFα (Hs01113624_g1), TGFβ1 (Hs00998133_m1), MMP1 (Hs00899658_m1), MMP2 (Hs01548727_m1), MMP9 (Hs00957562_m1). The relative gene expression levels were normalized to the expression of glyceraldehyde 3-phosphate dehydrogenase (GAPDH Hs99999905_m1), and the data are presented as the fold change using the formula 2^−ΔΔCt^, as recommended by the manufacturer (User Bulletin No. 2 P/N 4303859, Applied Biosystems). For each experimental time point, the gene expression of each marker was calculated relative to the control sample D3. The data are shown as the average of the duplicates ± SD and are representative of the three independent experiments.

### 4.8. Pro-Inflammatory Cytokines Detection

To evaluate the presence of the IL-6 proinflammatory cytokine released in the cell medium, ProQuantum high-sensitivity immunoassays (ThermoFisher Scientific, Monza, Italy) were performed according to the manufacturer’s instructions. Briefly, 2 μL of cell culture medium was incubated with the antibody-conjugate mixture to allow the formation of target-specific amplicons. Quantification was performed using a standard curve of IL-6 recombinant protein. The resulting signal was amplified using a 7500 Real-Time PCR instrument (Applied Biosystems, Life Technologies, Monza, Italy). Data were analyzed as concentration values and expressed as fold changes relative to control samples. Results are presented as the mean of duplicates ± SD from three independent experiments.

### 4.9. Statistical Analysis

All statistics were performed using Prism 5 (GraphPad, San Diego, CA, USA), applying one-way ANOVA and Tukey’s multiple-comparison test. The differences were considered significant at *p* < 0.05.

## 5. Conclusions

Overall, this study describes the establishment of a cost-effective, reproducible, and easily accessible 3D alveolosphere-based system that may represent a useful preliminary experimental framework for investigating lung epithelial senescence. The system exhibited features consistent with alveolar epithelial differentiation and developed multiple molecular and morphological changes commonly associated with cellular senescence following doxorubicin exposure. These characteristics support its use as an accessible starting point for investigating epithelial cell responses to senescence-inducing stimuli in a controlled 3D environment. In addition, the approach is technically straightforward, relatively low-cost, and potentially adaptable to larger experimental settings.

At the same time, this work should be regarded as a foundational step rather than a fully validated platform, and several aspects warrant further development. First, the model currently relies exclusively on alveolar epithelial cells and does not include other cellular components known to play a central role in IPF pathogenesis, such as fibroblasts, immune cells, and endothelial cells; their inclusion in future iterations of the model will be essential to more faithfully reproduce the multicellular complexity of the alveolar microenvironment and the cellular crosstalk underlying fibrotic remodeling. Second, evidence for pro-fibrotic signaling in this study was obtained primarily at the transcriptional level; future work incorporating protein-level and functional readouts will be needed to more directly assess extracellular matrix remodeling, ideally within longer-term or co-culture configurations better suited to capturing these slower processes. Third, while the senescence-associated phenotype observed here was supported by multiple converging lines of evidence, demonstrating its persistence following removal of the senescence-inducing stimulus will be an important next step to more firmly establish a stable senescent state, distinct from an acute, reversible stress response.

Despite these limitations, the present system provides a useful basis for further refinement and development. Future studies incorporating additional cell populations and functional validation will help determine the extent to which this approach can model selected aspects of the IPF microenvironment. More broadly, this work may support the development of increasingly sophisticated multicellular alveolosphere systems for studies of epithelial senescence and for the preliminary evaluation of candidate senotherapeutic compounds.

## Data Availability

The raw data supporting the conclusions of this article will be made available by the authors on request.
